# Improving soil carbon sequestration stability in *Siraitia grosvenorii* farmland through co-application of rice straw and its biochar

**DOI:** 10.3389/fpls.2024.1470486

**Published:** 2024-12-04

**Authors:** Xuehui Liu, Yu Yang, Yaqi Xie, Yicheng Zeng, Ke Li, Lening Hu

**Affiliations:** ^1^ Guangxi Key Laboratory of Environmental Processes and Remediation in Ecologically Fragile Regions, Ministry of Education, Guangxi Normal University, Guilin, China; ^2^ Key Laboratory of Ecology of Rare and Endangered Species and Environmental Protection, Ministry of Education, Guangxi Normal University, Guilin, China; ^3^ School of Civil Engineering, Guilin University of Technology, Guilin, China

**Keywords:** field experiment, microbial entropy, organic carbon components, soil amendments, soil metabolic entropy

## Abstract

**Introduction:**

Susbtantial agricultural wastes are produced globally which need urgent management policies. To explore the effective utilization of agricultural waste in enhancing soil quality and carbon sequestration capacity, straw and its biochar can be applied as soil ameliorants.

**Methods:**

This study was designed to investigate the impact of different return-to-field methods of rice straw on the transformation between different carbon components in the soil of *Siraitia grosvenori*i fields. We hypothesize that rice straw and its biochar, as soil amendments, can influence the transformation and cycling of different carbon components in the soil of *S. grosvenorii* fields through various return-to-field methods. Rice straw, rice straw biochar, and “rice straw + rice straw biochar” were applied as additives in a 2-year field experiment.

**Results:**

The results showed that the field application of rice straw and its biochar increased the content of soil organic carbon, the amount of organic carbon mineralization, particulate organic carbon, mineral-associated organic carbon, dissolved organic carbon, and readily oxidizable organic carbon content, while reducing the content of soil microbial biomass carbon. The combined application of rice straw and biochar in *S. grosvenorii* cultivation fields had a more significant effect on various soil carbon fractions compared to the use of either rice straw or biochar alone. The co-application of rice straw and its biochar to the soil increased the content of soil organic carbon by 117.4%, enhanced the mineralization of organic carbon by 100.0%, and reduced the content of soil microbial biomass carbon by 61.6%. The metabolic entropy and microbial entropy of rice straw and its biochar mixed application in the field were 5.2 and 0.18 times higher than of the control group, respectively.

**Discussion:**

In summary, the return of rice straw and biochar to the field improves soil structure and the content of recalcitrant organic carbon, providing a habitat for microorganisms, thereby promoting the stability and cycling of soil organic carbon.

## Graphical Abstract

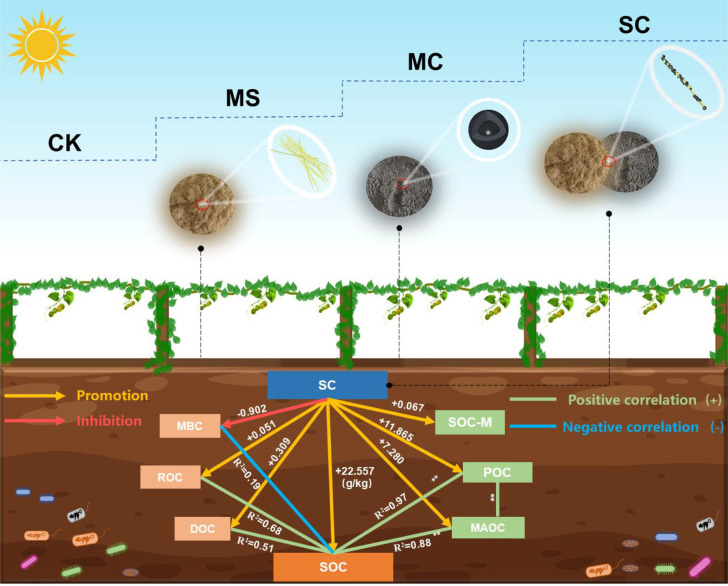

## 1 Introduction

*Siraitia grosvenorii* (namely Luo Han Guo), a climbing herb belonging to the family Cucurbittaceae. It has the effects of clearing the lungs and benefiting the throat, resolving phlegm and relieving cough, moistening the intestines and promoting bowel movements (Xiao, 2009). The main areas where it is cultivated are Guangdong, Guangxi, Guizhou, Hunan, and Jiangxi, among which cultivation in Guangxi is particularly intensive and has become a local specialty in Guangxi (Qi, 2012). For optimal growth, *S. grosvenorii* requires deep, humus-rich, loose, wet, and well-drained soil (Liang et al., 2019). However, in farmland used for the cultivation of *S. grosvenorii*, continuous cropping and the extensive use of chemical fertilizers and pesticides have led to issues such as decreased soil carbon pool activity, reduced organic matter content, and nutrient imbalances (Zhou et al., 2023).

Rice straw and its biochar are rich in C, N, P, K, and trace elements, all of which contribute to enhancing the soil physicochemical properties and improving its fertility (Li et al., 2023b). Research indicated that direct straw return to the field not only improved soil quality but also reduced chemical fertilizer usage, effectively decreasing the environmental pollution (Li et al., 2010; Yin et al., 2021). Moreover, the use of straw carbonization has attracted significant attention in agricultural and ecological research (Gao et al., 2019). Rice straw biochar, a carbon rich functional material, is prepared by pyrolysis in high-temperature and limited-oxygen environments; it has a large specific surface area, strong adsorption capacity, and stable properties (Kumar et al., 2017; Jia et al., 2024). Peng et al. (2023) revealed that the application of straw and biochar increased the soil carbon content. Similarly, Jia et al. (2016) demonstrated that the application of straw or straw biochar significantly elevated the levels of active organic carbon in soil.

Various fractions of organic carbon, including dissolved organic carbon (DOC), microbial biomass carbon (MBC), readily oxidizable organic carbon (ROC), particulate organic carbon (POC) and mineral-bound organic carbon (MAOC), serve as reliable indicators of the rate of decomposition of soil organic matter and the levels of microbial activity (Panchal et al., 2022). The mineralization of organic carbon reflects the decomposition process of soil organic carbon (SOC), which is closely related to the release of nutrients and CO_2_ emissions (Alvarez et al., 2018). Furthermore, POC and MAOC are excellent predictors of the response of SOC to perturbations in the external environment (Li, 2023). Consequently, this study selected these carbon fractions as indicators to assess the soil microbial activity and soil carbon pool stability.

To investigate the effective utilization of rice straw and its biochar in improving soil quality and carbon sequestration capacity. This study selects the agricultural soil from the high-yield cultivation demonstration area of *S. grosvenorii* in Shengli Village, Baoli Town, Yongfu County, Guilin City, as the research subject. Rice straw (MS), rice straw biochar (MC), and “rice straw + rice straw biochar” (SC) are used as additives. We hypothesize that the application of rice straw and rice straw biochar, either alone or in combination, can increase the soil organic carbon content. We also hypothesize that rice straw and its biochar can alter the composition of organic carbon in the soil, increase the proportion of recalcitrant organic carbon, and promote the long-term stability of soil carbon. By analyzing the impact of rice straw and its biochar on the mineralization of organic carbon and the content of carbon components in the soil of *S. grosvenorii* fields, we aim to provide a theoretical basis for studying the differences in the transformation of organic carbon in *S. grosvenorii* field soil and the potential for soil carbon sequestration.

## 2 Materials and methods

### 2.1 Study area and experimental design

**Figure 1:**
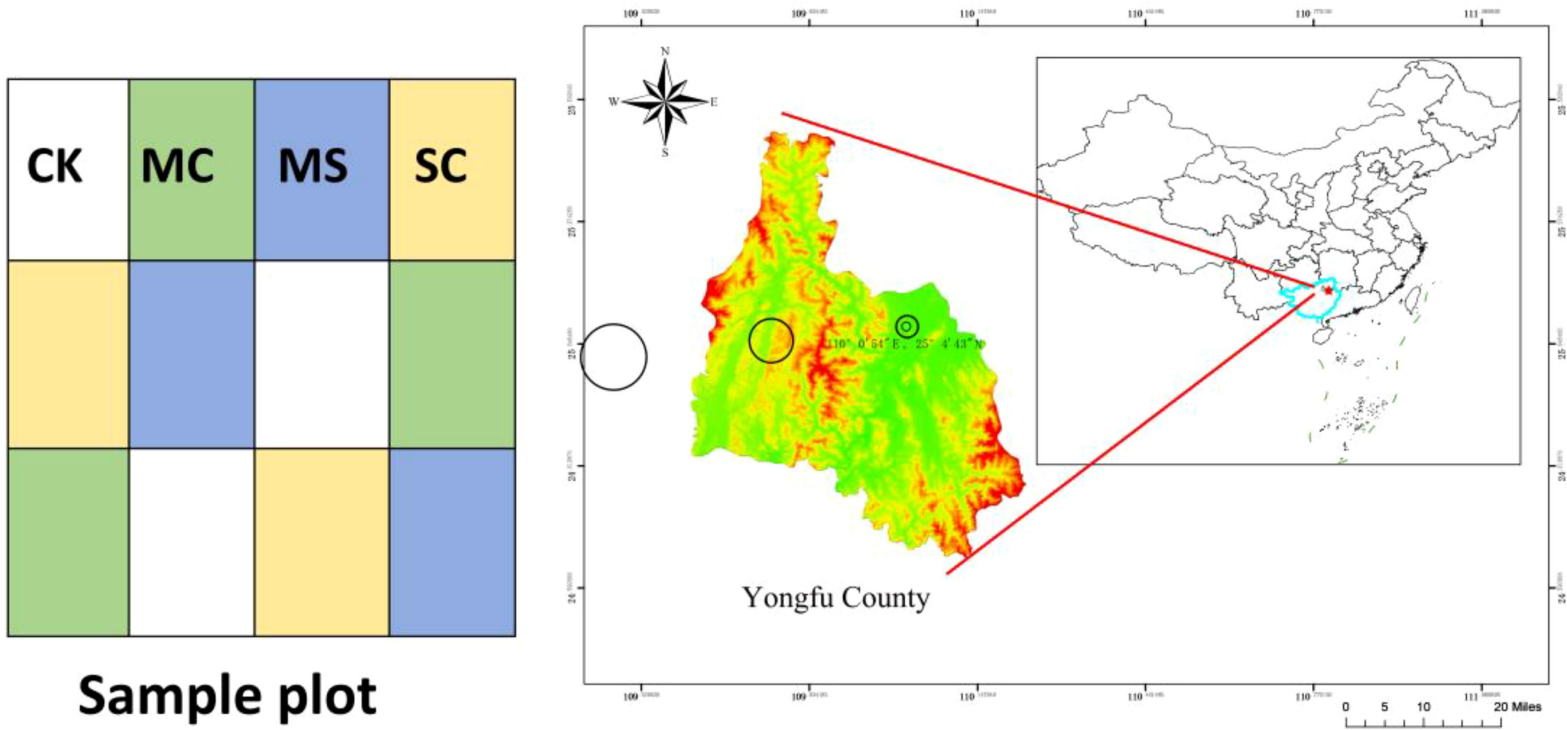
Soil sampling points and sample plot arrangement.

The research area was located in Guilin City, Guangxi Zhuang Autonomous Region. This area has a subtropical monsoon climate, with average rainfall of 1900–2000 mm annually. The sun shines year-round, offering over 1550 h of sunshine per annum, and the average temperature is 19°C (Huang, 2023). The sampling area was located in a demonstration area for the high-yield cultivation of *S. grosvenorii* in Shengli Village, Baoli Town, Yongfu County, Guilin City (25°4′43′′N, 110°0′54′′E). The soil under investigation was acidic yellow soil. The experimental area includes four treatments, with 3 replicates for each treatment. The treatment area was 2×1 m. The experimental design is shown in **Table 1**. The sampling points and experimental design layout are shown in **Figure 1**.

**Table 1:** Experimental design of *S. grosvenorii* test field.

Treatment	Imposer	Addition
CK	Nil	0
MS	Rice straw	9 kg/Sample plot
MC	Rice straw biochar	4.5 kg/Sample plot
SC	Rice straw + rice straw biochar	(4.5 + 2.25) kg/Sample plot

the rice straw harvested from the 1 hm^2^ field was the full amount (3582 kg·hm^-2^), and the rice straw biochar was calculated at 50% charcoal yield (1791 kg·hm^-2^). Under the premise of heavy application of basal fertilizer to *S. grosvenorii*, basically no fertilizer was applied during the seedling period; strong flower fertilizer was applied one to two times when a large number of blossoms came to seed from June to August, and 0.2% ternary compound fertilizer was used for drenching in water; strong fruit fertilizer was applied two to three times from August to October, and 0.2% compound fertilizer (15:5:25) was used for drenching in water.

### 2.2 Sample collection and processing

The soil samples were collected in September 2023. They were collected from the surface soil layer at depths ranging from 0 to 20 cm, resulting in a total of 12 soil samples. Prior to any testing, the soil samples were thoroughly cleaned to remove any animal and plant debris and gravel. Then, they were air-dried and sieved to a particle size of 2 mm to ensure consistent testing conditions. The soil background values of the tested *S. grosvenorii* farmland are shown in **Table 2**. The basic properties of the rice straw biochar are shown in **Table 3**.

**Table 2:** Soil background value of *S. grosvenorii* farmland.

pH	EC (mS·m^-1^)	SOC (g·kg^-1^)	AP (mg·kg^-1^)	AK (mg·kg^-1^)	CEC (cmol·kg^-1^)	NH_4_^+^-N (mg·kg^-1^)
5.7 ± 0.1	86.2 ± 4.3	18.3 ± 1.5	24.0 ± 0.1	88.7 ± 1.6	39.0 ± 2.4	208.2 ± 9.9

**Table 3:** Basic properties of rice straw and its biochar.

	pH	Elemental content(%)			
		C	N	K	P
rice straw	6.5	36.3	1.10	4.4	0.18
rice straw biochar	7.4	23.3	0.86	2.2	0.20

### 2.3 Assay method

The property determination of the rice straw and its biochar is as follows: pH was determined using a pH meter (water: biochar = 20:1; water: rice straw = 20:1). The C and N elemental content of rice straw and its biochar was determined using an elemental analyzer; the P and K elemental content in rice straw and its biochar was measured using Inductively Coupled Plasma Optical Emission Spectroscopy (ICP-OES).

The methods for determination soil indicators are as follows: The pH was determined using the pH meter method (water-to-soil ratio = 2.5:1), and the electrical conductivity (EC) was measured using the electrode method. Available potassium content (AK) was determined by the 1 mol·L^-1^ ammonium acetate extraction-flame photometer method. The available phosphorus (AP) content was determined by the 0.05 mol·L^-1^ HCl - 0.025 mol·L^-1^ H_2_SO_4_ method (Bao, 2000). The contents of ammonia nitrogen (NH_4_^+^-N) was assessed the potassium chloride solution extraction–spectrophotometry method (Yao, 2023). The cation exchange capacity (CEC) was determined by the cobalt hexamine trichloride extraction spectrophotometry (Chen et al., 2019).

The determination of active carbon components included the assessment of microbial biomass carbon (MBC) content using the chloroform fumigation extraction method. The content of organic carbon (SOC) was determined by potassium dichromate volumetric method and external heating method. The content of dissolved organic carbon (DOC) was determined by carbon automatic analyzer. Readily oxidizable organic carbon (ROC) content was determined by potassium permanganate oxidation method (Li, 2021).The particulate organic carbon (POC) and mineral-bound organic carbon (MAOC) were decomposed by sodium hexametaphosphate (Yao, 2023), and quantified using the potassium dichromate volumetric method with external heating. The amount of organic carbon mineralization was determined by BaCl_2_-HCl titration.


$$\begin{array}{c} \text{Organic carbon mineralization}({\text{mg}\cdot \text{kg}}^{-1})\\ =\{[\left({\text{V}}_{0}-\text{V}\right)\times \text{C}\times 0.022\times \left(22.4/44\right)\times 1000]\times 2\times 1000\}/\text{m} \end{array}$$

formula:


$${\text{V}}_{0}—\text{Volume of standard hydrochloric acid consumed in blank titration, mL};$$

V—Volume of standard hydrochloric acid consumed in sample titration, mL;

$$\text{C}—{\text{Concentration of standard hydrochloric acid, mol}\cdot \text{L}}^{-1};$$

$$\begin{array}{c} 0.022—\text{Molar mass of carbon dioxide }\left(1/2{\text{CO}}_{2}\right)\text{, M}\left(1/2{\text{CO}}_{2}\right)\\ =0.022{\text{ g}\cdot \text{mmol}}^{-1}; \end{array}$$

$$22.4\times 1000/44—{\text{Milliliters per gram of CO}}_{2}\text{in the standard state}$$

Microbial biomass entropy (qMBC) is the ratio of MBC to SOC, that is, qMBC = MBC/SOC (Wu et al., 2019). The soil metabolic quotient (qCO_2_) is the ratio of organic carbon mineralization to MBC, that is, qCO_2_ = organic carbon mineralization/MBC (Ji et al., 2020).

The organic carbon functional groups were analyzed by infrared spectroscopy, which was conducted on the soil surface functional groups (Ge et al., 2023). The soil was ground and sieved through 100 mesh and dried in an oven. A total of 1 mg of the soil sample was removed, and 200 mg of potassium bromide was added at a ratio of 1:200. Then, the sample was placed in an agate mortar and ground, and then the mixed powder was pressed with a tablet press at a pressure of 1.5 t and scanned by an infrared spectrometer.

### 2.4 Data processing

The experimental data were analyzed and sorted by Excel 2016. Origin 2024 and IBM SPSS Statistics 22 software were used for statistical analysis and visualization of the data. Pearson’s correlation analysis was employed to investigate the relationship between the soil carbon component content and various physicochemical properties in the *S. grosvenorii* farmland. The significance level was set to *P<* 0.05. Random forest modeling was used to analyze the data using the R language. Data are presented as mean ± standard deviation.

## 3 Result

### 3.1 Effects of the rice straw and its biochar returned to the field on the physicochemical properties of soil in *Siraitia grosv*enorii farmland

**Figure 2:**
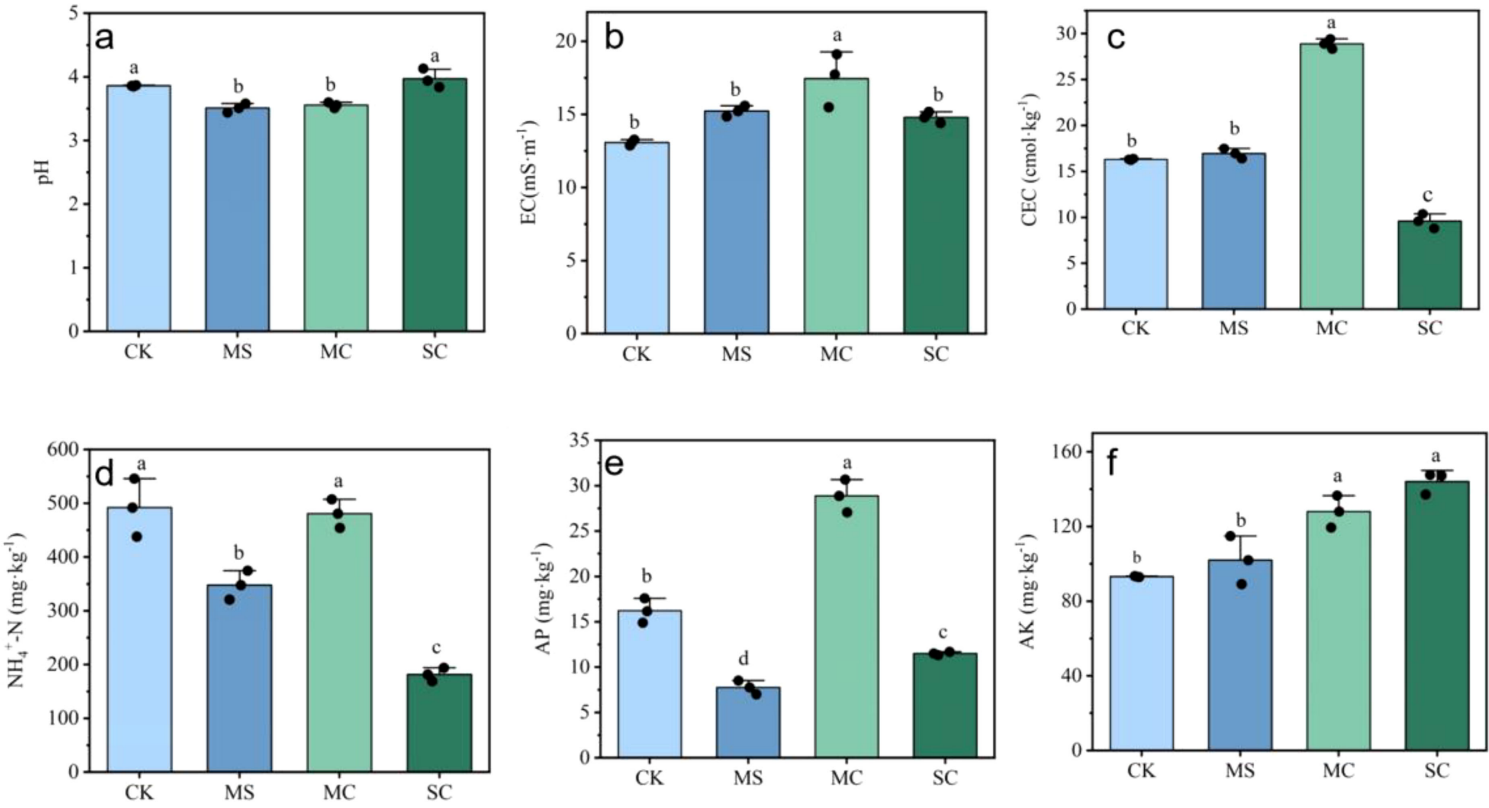
Effects of application of rice straw and its biochar return on physicochemical properties of soil in *S. grosvenorii* farmland [**(A)** pH; **(B)** electrical conductivity (EC) content; **(C)** cation exchange capacity (CEC) content; **(D)** ammonia nitrogen (NH_4_^+^-N) content; **(E)** available phosphorus content (AP); **(F)** available potassium content (AK)]. Lowercase letters indicate the significance of the data, with different lowercase letters representing significant differences.

The application of rice straw and its biochar increased the EC and AK contents and decreased the NH_4_^+^-N content of the soil of the *S. grosvenorii* farmland (**Figures 2B, D, F**). MC had the greatest effect on EC, which increased by 33.3%, and the least effect on NH_4_^+^-N, which decreased by 2.3%. SC had the greatest effect on AK, with an increase of 50.8 mg·kg^-1^ (AK). The MS and SC treatments reduced the AP content, and the AP content of the *S. grosvenorii* farmland soil decreased the most by 8.5 mg·kg^-1^ (**Figure 2E**). When compared with the CK, the MS and MC treatments decreased the soil pH and increased the CEC, while the SC treatment increased the soil pH and decreased the CEC (**Figures 2A, C**).

### 3.2 Effects of the rice straw and its biochar return on soil organic carbon content and its mineralization

**Figure 3:**
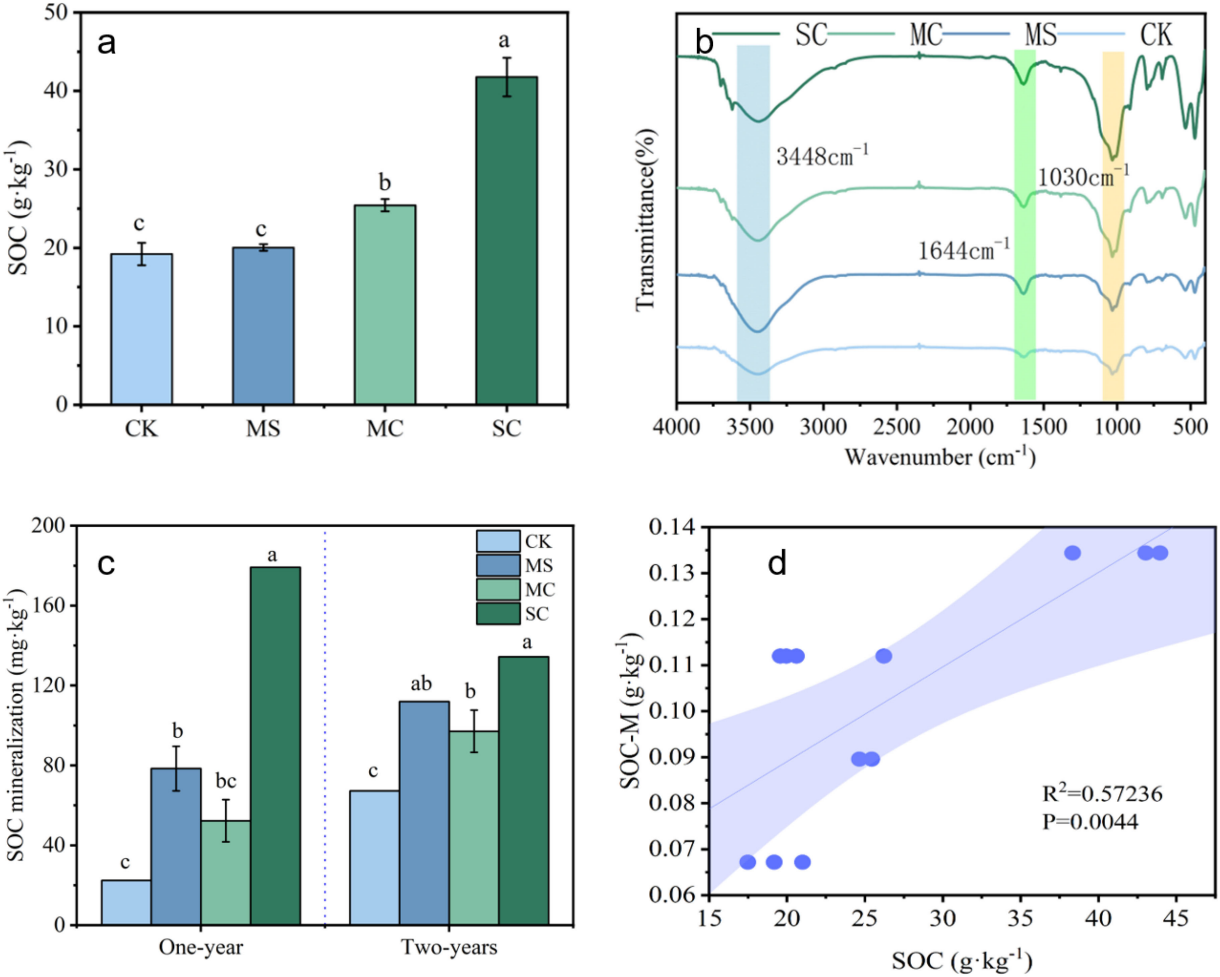
Effect of rice straw and its biochar return on organic carbon content and mineralization of soil in *S. grosvenorii* farmland [**(A)** organic carbon (SOC) content; **(B)** soil infrared spectra; **(C)** soil organic carbon mineralization; **(D)** effect of organic carbon mineralization and organic carbon content].

Application of rice straw and its biochar increased the soil organic carbon (SOC) content, and SC increased the SOC content of the soil of the *S. grosvenorii* farmland the most, by 22.6 g·kg^-1^ (**Figure 3A**). The shapes of the soil infrared spectra under each treatment were basically the same, with similar characteristic peaks. The soil had obvious characteristic peaks at 3448, 1644, and 1030 cm^-1^ were assigned to O-H/N-H vibration peak, the C=C of aromatic carbon and the C=O vibration in -COO-, and Si-O-Si bonds, respectively. The application of rice straw and its biochar increased the relative intensity of the absorption peaks at 3448, 1644, and 1030 cm^-1^ in the soil of *S. grosvenorii* farmland, in which the SC treatment presented the greatest effect (**Figure 3B**).

The application of rice straw and its biological carbon increased the amount of organic carbon mineralization (SOC-M) in the *S. grosvenorii* farmland. Among the treatments, the SC had the most significant effect on soil mineralization, with the mineralization amounts in the first and second years being 8 and 2 times greater than that of those in CK, respectively. Conversely, the MC had the smallest effect, with an increase of 29.9 mg·kg^-1^ in the first and second years when compared with the CK. For the first year of soil organic carbon mineralization in *S. grosvenorii* farmland, the SC treatment reduced the amount of mineralization by 44.8 mg·kg^-1^. MS and MC increased the mineralization of organic carbon, with MS showing the smallest increase at 33.6 mg·kg^-1^ (**Figure 3C**). According to linear correlation analysis, the amount of organic carbon mineralization showed a positive correlation with SOC content (**Figure 3D**).

As shown in **Table 4**, rice straw and its biochar returned to the field increased metabolic entropy of the soil in the *S. grosvenorii* farmland. Compared with CK, the metabolic entropy of the MS, MC, and SC treatments was 4.2, 4.3, and 5.2 times that of the CK, respectively. SC had the greatest enhancement of soil metabolic entropy and MS had the least enhancement of soil metabolic entropy.

**Table 4:** Effect of rice straw and its biochar return on soil metabolic entropy (qCO_2_).

	CK	MS	MC	SC
qCO_2_	0.046 ± 0.003	0.191 ± 0.012	0.198 ± 0.024	0.239 ± 0.002

### 3.3 Effects of rice straw and its biochar return on particulate organic carbon and mineral-bound organic carbon in *Siraitia grosvenorii* farmland soil

**Figure 4:**
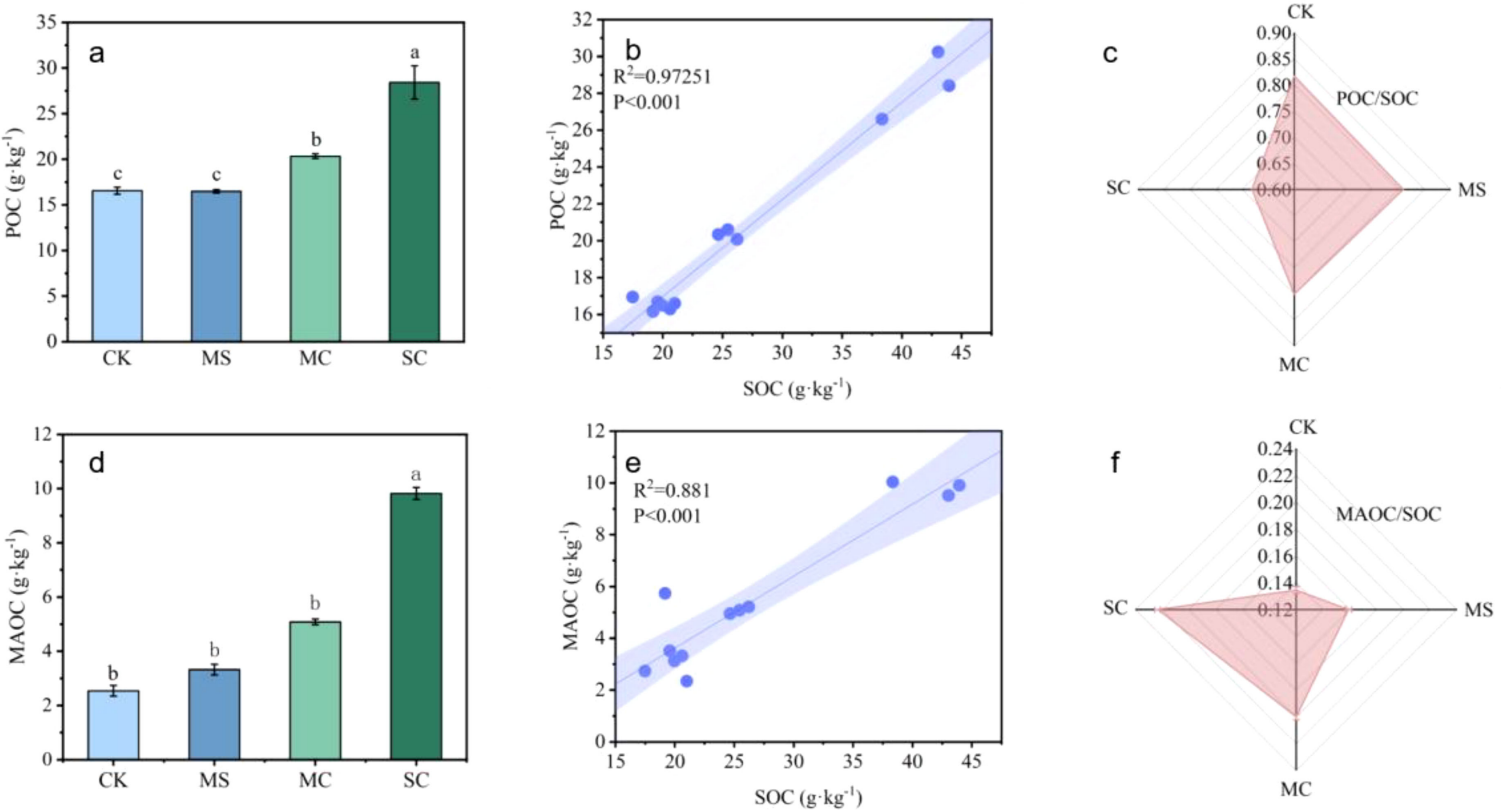
Effect of rice straw and its biochar return on POC and MAOC content of *S. grosvenorii* farmland [**(A)** content of particulate organic carbon (POC); **(B)** correlation between POC and SOC; **(C)** POC/SOC values; **(D)** content of mineral bound organic carbon (MAOC); **(E)** correlation between MAOC and SOC; **(F)** MAOC/SOC values]. Lowercase letters indicate the significance of the data, with different lowercase letters representing significant differences.

Based on soil particle size, organic carbon was classified as particulate organic carbon (POC) and mineral bound organic carbon (MAOC), respectively. When compared with the CK, the application of rice straw and its biochar increased the content of MAOC in the soil of *S. grosvenorii* farmland. The SC treatment increased the maximum MAOC content in soil, with an increase of 7.3 g·kg^-1^; the MC treatment increased the MAOC content to 2.5 g·kg^-1^. The MS treatment reduced the POC content by 3.9%, and the MC and SC treatments increased the POC content by 22.9% and 71.7%, respectively (**Figures 4A, D**). According to the linear correlation analysis, POC, MAOC content and SOC content showed a positive correlation (**Figures 4B, E**). The application of rice straw and its biochar decreased the content of POC in soil organic carbon and increased the content of MAOC in soil organic carbon in *S. grosvenorii* farmland. The SC had the greatest effect on the POC and MAOC contents in the organic carbon; the POC/SOC decreased by 0.136, and the MAOC/SOC increased by 0.088 (**Figures 4C, F**).

### 3.4 Effects of rice straw and its biochar return on the active organic carbon content of soil in *Siraitia grosvenorii* farmland

**Figure 5:**
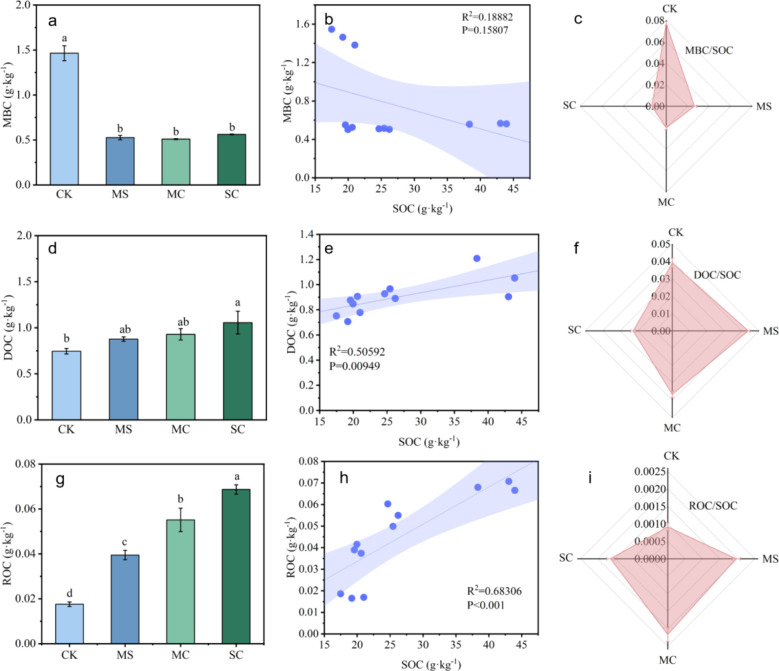
Effects of rice straw and its biochar on soil active organic carbon in *S. grosvenorii* farmland [**(A)** microbial bulk carbon (MBC) content; **(B)** relationship between MBC content and SOC content; **(C)** MBC/SOC value (microbial entropy); **(D)** dissolved organic carbon (DOC) content; **(E)** relationship between DOC content and SOC content; **(F)** DOC/SOC value; **(G)** readily oxidizable organic carbon (ROC) content; **(H)** ROC content and SOC content; **(I)** ROC/SOC value). Lowercase letters indicate the significance of the data, with different lowercase letters representing significant differences.

Active organic carbon consists of microbial biomass carbon (MBC), dissolved organic carbon (DOC), and readily oxidizable organic carbon (ROC). Compared with CK, the treatments including MS, MC, and SC led to a reduction in MBC content by approximately 0.90 g·kg^-1^ (**Figure 5A**). MBC/SOC ratio represents the microbial entropy of the soil, indicating microbial efficiency in organic carbon utilization. Compared with CK, microbial entropy in MS, MC, and SC treatments decreased by 65.4%, 68.9%, and 82.0%, respectively. Among all treatments, SC had the most pronounced effect on microbial entropy, showing the highest reduction (**Figure 5C**). According to the linear correlation analysis, MBC and SOC content showed a negative correlation (**Figure 5B**). The application of rice straw and its biochar significantly increased the content of DOC and ROC; specifically, the SC treatment led to increases of 0.309 g·kg^-1^ in DOC and 0.051 g·kg^-1^ in ROC (**Figures 5D, G**). Based on the linear correlation analysis of DOC, ROC content showed a positive correlation with SOC content (**Figures 5E, H**). Compared with the CK, the application of rice straw and its biochar increased the proportion of ROC in SOC, while the ROC/SOC ratio remained low, with values below 0.003 (**Figure 5I**). The MS treatment increased the proportion of DOC in the SOC by 0.005 (**Figure 5F**).

### 3.5 Analysis of relationship

**Figure 6:**
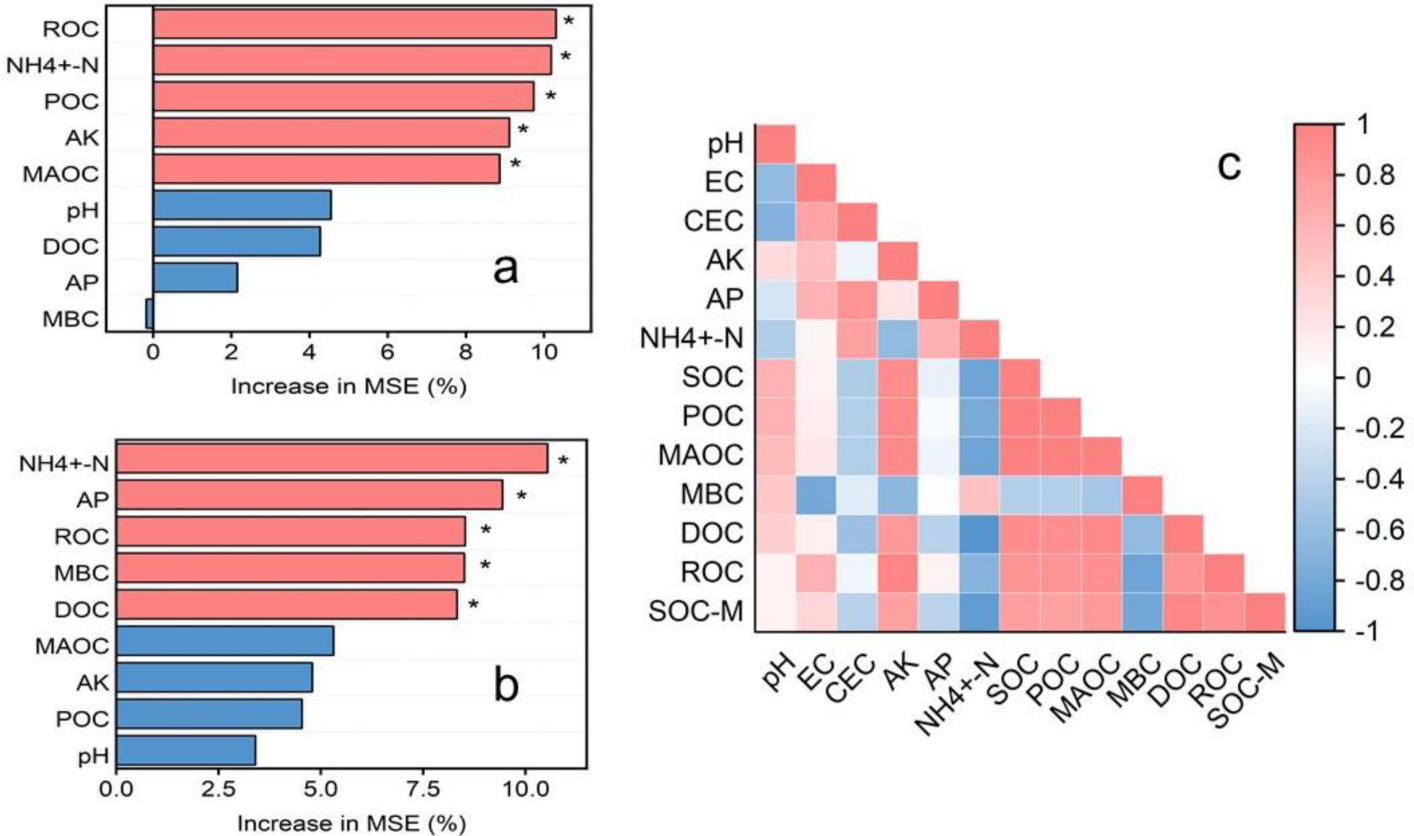
Correlation analysis [**(A)** random forests, importance of carbon fractions and nutrients as drivers for organic carbon (SOC); **(B)** random forests, importance of carbon fractions and nutrients as drivers for organic carbon mineralization (SOC-M)] **(C)** heat map of correlation of organic carbon and its carbon fractions with physical-chemical properties). *P≤ 0.05.

According to the random forest model analysis, among the nine features of the RF (SOC) model (**Figure 6A**), ROC, NH_4_^+^-N, POC, AK, and MOAC rank as the top five in importance, with ROC and NH_4_^+^-N each contributing approximately 10%. Among the nine features in the RF (SOC-M) model (**Figure 6B**), the importance of NH_4_^+^-N, AP, ROC, MBC, DOC, MAOC, AK, POC, and pH follows the order of NH_4_^+^-N > AP >ROC = MBC > DOC, with NH_4_^+^-N accounting for approximately 10%, AP for 9%, and ROC, MBC, and DOC each contributing between 8% and 9%. NH_4_^+^-N and ROC showed importance for both SOC and SOC-M. As shown in the correlation heat map (**Figure 6C**), SOC exhibited a significant positive correlation with both POC and MAOC (*P* ≤ 0.01), and POC was also significant positive correlation with MAOC (*P* ≤ 0.01). MBC was negatively correlated with SOC, DOC, and ROC. DOC showed a negative correlation with NH_4_^+^-N (*P* ≤ 0.05) and a positive correlation with SOC-M (*P* ≤ 0.05), while ROC was positively correlation with AK (*P* ≤ 0.05).

## 4 Discussion

### 4.1 Effects of returning rice straw and its biochar to the field on the physical and chemical properties of soil

The physicochemical properties of the soil are important indicators of soil health (Huang et al., 2017). Here, the application of rice straw and its biochar increased the available potassium (AK) content and electrical conductivity in the soil of *S. grosvenorii* farmland, while reducing the ammonia nitrogen content. The increase in the AK content was mainly due to the high potassium content in the rice straw and its biochar (Xia et al., 2022), and biochar has been shown to significantly enhance the abundance of silicate bacteria, which aids in the activation of soil mineral potassium (Jiang et al., 2016). Therefore, the AK content under the MC treatment was higher than that under the MS treatment, with the SC treatment showing the greatest effect on increasing AK content. Furthermore, while the application of rice straw and its biochar reduced the content of soil ammonia nitrogen levels, the rice straw biochar returned to the field had a less pronounced effect on reducing these nitrogen contents. The decrease in ammonia nitrogen levels in the field where rice straw and its biochar were applied is primarily attributed to the increased C/N ratio of the soil, which promoted the conversion of available nitrogen into microbial biomass nitrogen or organic nitrogen (Wen, 2019). Rice straw biochar is linked to the minimal reduction in ammonia nitrogen content, because it can increase the adsorption capacity of inorganic nitrogen, slow down the desorption or isolation of compounds, curbs soil nitrogen mineralization, and reduce nitrogen leaching (Liu et al., 2020a). The return of rice straw to the field, compared to the application of biochar, can decompose and produce a large number of alkali ions, thereby increasing the cation exchange capacity of soil (Uchimiya et al., 2011; Yuan et al., 2011). However, the SC treatment reduced the cation exchange capacity of soil, a result that aligns with research by Pignatello. Here, the organic carbon content associated with the SC treatment was much higher than those with the MS and MC treatments, while Pignatello suggested that organic matter can enrich humic and fulvic acids in the soil, clogging the inherent pores of the biochar, preventing it from proceeding to the next step of physical adsorption (Kwon and Pignatello, 2005).When compared with CK, both the MS and SC treatments reduced the available phosphorus (AP) content. Pearson’s correlation analysis revealed a positive correlation between AP and cation exchange capacity. The addition of rice straw altered the activity of cations in the soil, thus increasing, the adsorption of soil phosphorus or reducing its desorption, which weakened the effectiveness of the phosphorus (Yang and Lu, 2022).

### 4.2 Effects of rice straw and its biochar on soil organic carbon and its functional groups

Soil organic carbon (SOC) is a crucial component of the soil carbon pool, serving as a vital indicator for assessing soil quality and significantly impacting the carbon cycle balance within ecosystems (Lal, 2004; Sheng et al., 2018). Compared with the CK treatment, the MS, MC, and SC treatments increased the soil organic carbon content, in the following order: SC > MC > MS > CK. Thus, rice straw and its biochar can supplement the organic carbon content of the soil. Rich carbon sources can improve soil microbial absorption and utilization, improve the number and activity of microorganisms, and release more organic carbon (Fan, 2023). Biochar, owing to its porous network, forms micro-aggregates with soil minerals, which physically protect SOC, enhancing its stability (Weng et al., 2017). This indicates that the application of rice straw and its biochar may lead to an increase in the SOC content in experiments involving long-term observations, consistent with the results of research by Singh and others (Singh et al., 2019).

In this study, the main organic carbon functional groups of the *S. grosvenorii* farmland soil were aromatic carbon, alcohols, phenols, and silicate minerals. Aromatic carbon is one of the organic carbon types that does not readily decompose, so it can be selectively retained in the soil, and organic carbon in the soil increases the intensity of aromatic carbon during the decomposition process (Guo et al., 2013; Li et al., 2022). Both the rice straw and the rice straw biochar contain components with high chemical stability, which can increase the aromatic carbon in the SOC by promoting the accumulation of resistant compounds in the soil. In this study, the application of rice straw and its biochar increased the organic carbon content in the soil. Over time, a large amount of unstable carbon probably gradually decomposed, and more stable carbon, such as aromatic carbon, accumulated (Ge et al., 2023). The SC treatment increased the SOC content, which promoted the accumulation of more stable carbon (aromatic carbon) in the *S. grosvenori* soil.

### 4.3 Effects of returning rice straw and its biochar to the field on soil carbon fractions

The particulate organic carbon (POC) form is mainly produced by the activities of plant, including a large number of plant carbon sources; and mineral-bound organic carbon (MAOC) is produced by microbial activity, with microbial carbon accounting for a large proportion of the total (Cambardella and Elliott, 1992). The results of a meta-analysis by Zhang showed that the effect of the exogenous organic matter input on the MAOC was greater than that on the POC (Zhang et al., 2022). In this study, the effects of the rice straw and its biochar on the MAOC and POC were similar to these findings. The application of rice straw and its biochar increased the content of POC and MAOC in the soil mainly because the rice straw and its biochar provided abundant carbon sources for the SOC (Simonetti et al., 2016). A positive correlation among POC, MAOC, and SOC was identified. The application of rice straw and its biochar increased the SOC content in which *S. grosvenorii* was grown. Therefore, it also increased the contents of POC and MAOC; however, the SC treatment had the most pronounced effects on the contents of POC and MAOC in the *S. grosvenorii* soil.

Microbial biomass carbon (MBC), dissolved organic carbon (DOC), and readily oxidizable organic carbon (ROC) have the characteristics of rapid movement, poor stability, and easy oxidation and decomposition (Bongiorno et al., 2019). In this study, the application of rice straw and its biochar increased the contents of DOC and ROC in the soil of farmland in which *S. grosvenorii* was cultivated. Notably, the SC treatment had the greatest effect. The correlation analysis, also revealed positive correlations between DOC and SOC, and between ROC and SOC. The effects of the rice straw and its biochar on DOC and ROC were the same as that on the SOC content. However, the application of rice straw and its biochar reduced the MBC content of the soil in which *S. grosvenorii* was grown. The MS and MC treatments decreased the MBC by approximately 64.0%, and the SC treatment decreased it by 61.0%. Regarding an explanation for the decrease in MBC content, this may have occurred because, after the application of rice straw and its biochar to acidic soil, their strong adsorption and loose porous nature caused some soil microorganisms to adhere to the pores, reducing the contact between microorganisms and organic matter (Niu, 2017). Additionally, when nitrogen becomes the limiting factor in the supply of soil nutrients, the metabolic activity of microorganisms will correspondingly decrease, thereby reducing the MBC content (Wang et al., 2010). In this study, no chemical fertilizers were applied to the agricultural soil at the time of *S. grosvenorii* maturity, leading to a shortage of nitrogen supply and inhibiting microorganism growth.

Soil microbial entropy (qMBC) is an index that is used to evaluate the accumulation or loss of organic carbon in the soil. The higher the qMBC, the higher the SOC activity (Li et al., 2023a). In this study, the application of rice straw and its biochar reduced the qMBC of soil in which *S. grosvenorii* was cultivated, and the qMBC of the SC treatment was the lowest. The results showed that the application of rice straw and its biochar reduced the organic carbon activity of the soil and improved the stability of the soil carbon pool. Among the treatment, “rice straw + rice straw biochar” was more conducive to improving soil carbon pool stability.

### 4.4 Effects of rice straw and its biochar on the mineralization of soil organic carbon

Soil respiration is used to evaluate the total activity of soil microorganisms and soil fertility, which can reflect the intensity of biological activities in the soil and soil material and energy conversion (Hang, 2023). Here, the application of rice straw and its biochar increased the mineralization of SOC in the *S. grosvenorii* farmland in the following order: SC > MS > MC > CK. The primary cause of this enhancement can be attributed to the introduction of rice straw and its biochar, which stimulated soil microorganisms and consequently boosted the soil respiration rate in the *S. grosvenorii* field (Hang, 2023). The application of rice straw biochar had little effect on the mineralization of soil organic carbon, which was similar to the results of reported by Zhang et al. (2019). Meanwhile, the MS and SC treatments had a large influence on the mineralization of soil organic carbon. First, the rapid decomposition of the active organic carbon in the rice straw directly increased the intensity of soil respiration (Zhang et al., 2012). Second, straw decomposition produces humus, which can provide a source of carbon for microbial growth and enhances the activity of microbes in the soil (Liu et al., 2020b).

Soil metabolic entropy can reflect the amount of carbon consumed by microbial respiration. Thus, the lower the soil metabolic entropy, the higher the efficiency of microbial carbon utilization (Duan et al., 2019). In this study, all three returning methods increased the metabolic entropy of the soil, but the SC treatment increased it the most, indicating that this treatment improved the efficiency of microbial carbon utilization.

### 4.5 The ecological impact of returning rice straw and its biochar to the field

Rice straw and its biochar, as soil amendments, can improve soil structure, increase soil fertility, and enhance the soil’s capacity to sequester carbon. Here, the application of rice straw and its biochar to the field increased the content of refractory organic carbon in the soil to some extent (e.g., aromatic carbon), promoting the fixation of stable soil carbon and helping to reduce greenhouse gas emissions from the soil. At the same time, the porous structure of rice straw and its biochar can provide a habitat for microorganisms, increasing microbial diversity and activity, and affecting soil nutrient cycling. However, insufficient soil nitrogen supply can inhibit microorganisms’ growth and activity. The use of rice straw and its biochar can reduce the use of chemical fertilizers, decrease the environmental impact of agricultural production, and at the same time mitigate the problem of environmental pollution in the form of agricultural waste. Here, the effect of field application of rice straw and its biochar in combination was superior to the application of either of these alone. This combination can promote the efficient use of rice straw and reduce some of the costs of producing biochar.

## 5 Conclusion

(1) The combined application of rice straw and its biochar greatly enhanced the accumulation of potassium in the soil. Specifically, the AK content of soil in this farmland, upon treatment with a combination of rice straw and its biochar, increased to levels that were 5.77 and 1.46 times higher than those achieved by rice straw and rice straw biochar alone, respectively.

(2) The application of rice straw and its biochar in combination also increased the soil organic carbon content in *S. grosvenorii* farmland more than the application of either of these alone. The application of rice straw and rice straw biochar was more conducive to POC and MAOC accumulation, and had a greater impact on MAOC than on POC. This combination also increased the mineralization and decreased the MBC content of the *S. grosvenorii* farmland soil, which was more conducive to improving the stability of the carbon pool in this soil.

In summary, the application of rice straw and rice straw biochar in combination is not only beneficial to soil carbon pool stability in *S. grosvenorii* farmland, but also achieves efficient utilization of rice straw and reduces the cost of preparing biochar. The application of rice straw and its biochar reduced the activity of SOC and the efficiency of microbial utilization of carbon in the field where *S. grosvenorii* was cultivated, and the effect of this mixed application was greater than that of the application of either of these agents alone.

## Data Availability

The original contributions presented in the study are included in the article/supplementary material. Further inquiries can be directed to the corresponding authors.
